# Fine-Tuning Roles of *Osa-miR159a* in Rice Immunity Against *Magnaporthe oryzae* and Development

**DOI:** 10.1186/s12284-021-00469-w

**Published:** 2021-03-06

**Authors:** Jin-Feng Chen, Zhi-Xue Zhao, Yan Li, Ting-Ting Li, Yong Zhu, Xue-Mei Yang, Shi-Xin Zhou, He Wang, Ji-Qun Zhao, Mei Pu, Hui Feng, Jing Fan, Ji-Wei Zhang, Yan-Yan Huang, Wen-Ming Wang

**Affiliations:** Rice Research Institute and Key Lab for Major Crop Diseases, Sichuan Agricultural University, Chengdu, 611130 China

**Keywords:** microRNA, *Osa-miR159a*, Short tandem target mimic, *Magnaporthe oryzae*, Rice blast disease

## Abstract

**Background:**

Rice blast caused by *Magnaporthe oryzae* is one of the most destructive diseases of rice. An increasing number of microRNAs (miRNAs) have been reported to fine-tune rice immunity against *M. oryzae* and coordinate with growth and development.

**Results:**

Here, we showed that rice microRNA159a (*Osa-miR159a*) played a positive role in rice resistance to *M. oryzae*. The expression of *Osa-miR159a* was suppressed in a susceptible accession at 12, 24, and 48 h post-inoculation (hpi); it was upregulated in a resistant accession of *M. oryzae* at 24 hpi. The transgenic rice lines overexpressing *Osa-miR159a* were highly resistant to *M. oryzae*. In contrast, the transgenic lines expressing a short tandem target mimic (STTM) to block *Osa-miR159a* showed enhanced susceptibility. Knockout mutations of the target genes of *Osa-miR159a*, including *OsGAMYB*, *OsGAMYBL*, and *OsZF*, led to resistance to *M. oryzae*. Alteration of the expression of *Osa-miR159a* impacted yield traits including pollen and grain development.

**Conclusions:**

Our results indicated that *Osa-miR159a* positively regulated rice immunity against *M. oryzae* by downregulating its target genes. Proper expression of *Osa-miR159a* was critical for coordinating rice blast resistance with grain development.

**Supplementary Information:**

The online version contains supplementary material available at 10.1186/s12284-021-00469-w.

## Background

Plant microRNAs (miRNAs) act as fine-tuning regulators and play regulatory roles in gene expression via cleavage, translational inhibition, or DNA methylation of target sites with sequences complementary to the miRNAs (Song et al. 2019). To date, more than 38,000 mature miRNAs have been reported in miRBase (http://www.mirbase.org/). Among them, 757 mature miRNAs have been identified in rice.

miRNAs play major roles in many biological processes, including functions related to response to biotic and abiotic stressors (Jones-Rhoades et al. 2006; Miura et al. 2010; Yan et al. 2016; Li et al. 2019b). Functional studies of many miRNAs have been done on *Arabidopsis* and rice. For example, knockout of *miR396ef* results in increased grain yield in rice via increasing grain size and panicle branching due to disinhibition of the expression of *OsGRF4* and *OsGRF6*, which are the target genes of *miR396* (Zhang et al. 2019; Miao et al. 2020). The overexpression of *miR1873* results in defects in yield traits by repressing its target gene *LOC_Os05g01790* (Zhou et al. 2020). *miR535* is highly expressed in rice panicles. Enhanced accumulation of *miR535* reduces plant height, modifies panicle architecture, and increases the grain length by regulating *OsSPLs* (Sun et al. 2019). *miR167* regulates stamen and gynoecium development in immature flowers by regulating the target genes *ARF6* and *ARF8* in *Arabidopsis* (Wu et al. 2006). Increasing evidence shows that miRNAs are involved in rice immunity against *Magnaporthe oryzae*. For example, overexpression of *miR1873* enhanced the susceptibility of rice to *M. oryzae* by regulating its target gene *LOC_Os05g01790* (Zhou et al. 2020). In addition, *miR396*, *miR169*, *miR164a*, *miR319b*, and *miR167d* negatively regulate immunity against *M. oryzae* in rice (Li et al. 2017a; Wang et al. 2018; Zhang et al. 2018; Chandran et al. 2019; Zhao et al. 2019), whereas *miR398b*, *miR160a*, *miR166k-miR166h*, *miR7695*, and *miR162a* positively regulate the response against *M. oryzae* in rice (Achard et al. 2004; Salvador-Guirao et al. 2018; Li et al. 2019a; Li et al. 2019b; Quoc et al. 2019; Li et al. 2020a).

The highly conserved and abundant 21 nucleotide (nt) miRNAs, *miR159* and *miR319*, share a sequence identity of 17 out of 21 nt matching with that of *Arabidopsis* (Palatnik et al. 2007). However, *miR159* and *miR319* function differently through distinct target genes. *miR319* targets *PROLIFERATING CELL NUCLEAR ANTIGEN BINDING FACTOR* (*TCP*) transcription factor genes, which control leaf shape (Schwab et al. 2005; Palatnik et al. 2007), while *miR159* targets a family of genes encoding R2R3 *MYB* transcription factors, which are referred to as GAMYBs or GAMYB-likes (GAMYBLs), and function in flowering and male fertility (Millar et al. 2019). The *miR159*-*GAMYB* regulatory module has been identified in major land plants, including *Arabidopsis* and rice. This module has been reported to act in growth and development. For example, in *Arabidopsis*, *miR159* suppresses the expression of *MYB33* and *MYB65* to regulate plant growth and development. *mir159a mir159b* (*mir159ab*) double mutant displays severe growth and developmental defects including curled rosette leaves and stunted plant height (Allen et al. 2007). These phenotypes may be due to the failure to suppress the expression of *MYB33* and *MYB65* by *miR159* (Allen et al. 2007; Alonso-Peral et al. 2010). Also, up-regulation of *miR159* impacts anther development and delays flowering. Moreover, *miR159* plays a crucial role in pollen fertility, and pollen-carried *miR159* abolishes the expression of *MYB33* and *MYB65* in the central cell after fertilization, promoting endosperm nuclear division and seed development (Zhao et al. 2018). *miR159* regulates flowering time and development during the short-day photoperiod by directly cleaving the mRNA of *GAMYB*-related genes that encode proteins involved in GA-promoted activation of LEAFY (Achard et al. 2004). In addition, *miR159-MYB33* functions as a modifier of vegetative phase change in *Arabidopsis* (Guo et al. 2017).

The rice genome contains six *Osa-miR159* genes generating five mature isoforms: *Osa-miR159a, Osa-miR159b*, *Osa-miR159c*, *Osa-miR159d*, *Osa-miR159e*, and *Osa-miR159f*. These isoforms mediate mRNA cleavage of three genes, *GAMYB* (*LOC_Os01g59660*), *GAMYBL* (*LOC_Os06g40330*), and *ZF* (encoding a C3HC4-type domain-containing zinc finger protein, *LOC_Os10g05230*). *GAMYB* has been shown to function in rice development. For example, *miR159-GAMYB* modulates the expression of gibberellic acid (GA)/abscisic acid (ABA)-related genes to maintain the energy supply and enhance developmental processes in Wuxiang S, a photo-thermosensitive genic male sterile rice line (Zhang et al. 2016a). In addition, a few studies show that *miR159* functions in plant immunity. For example, in cotton, *miR159* and *miR166* are increased in response to infection by *Verticillium dahliae*, and are exported to the fungal hyphae to silence the target genes *Ca*^*2+*^*-dependent cysteine protease* (*Clp-1*) and an *isotrichodermin C-15 hydroxylase* (*HiC-15*), which are essential for fungal virulence (Zhang et al. 2016b). In *Lilium regale*, *Ire-miR159a* positively regulates the plant’s resistance to grey mold caused by *Botrytis elliptica* by repressing the expression of its target gene *LrGAMYB* (Gao et al. 2020). In a previous study, we found that *Osa-miR159a* was differentially accumulated in susceptible and resistant accessions of rice (Li et al. 2014). However, its function in rice immunity has not been characterized.

In this study, we further functionally characterized *Osa-miR159a*. To accomplish this, we obtained the transgenic lines overexpressing *Osa-miR159a* (OX159) and the suppressed expression of *Osa-miR159a* (STTM159) through short tandem target mimic (STTM), which is an effective method to block mature miRNA binding to target sites of the target genes (Yan et al. 2012). In addition, we also constructed the knockout transgenic lines of *GAMYB*, *GAMYBL*, and *ZF* using the CRISPR/Cas9 method. Then, these transgenic lines were subjected to a *M. oryzae* disease assay and the phenotypic assay. We found that *Osa-miR159a* acts as a positive regulator in rice resistance to *M. oryzae* by suppressing *GAMYB*, *GAMYBL*, and *ZF*. It also impacted reproductive development in rice. Therefore, proper accumulation of *Osa-miR159a* was necessary to fine-tune the development and resistance to *M. oryzae* in rice.

## Results

### *Osa-miR159a* is Responsive to *M. oryzae* Infection

**Fig. 1:**
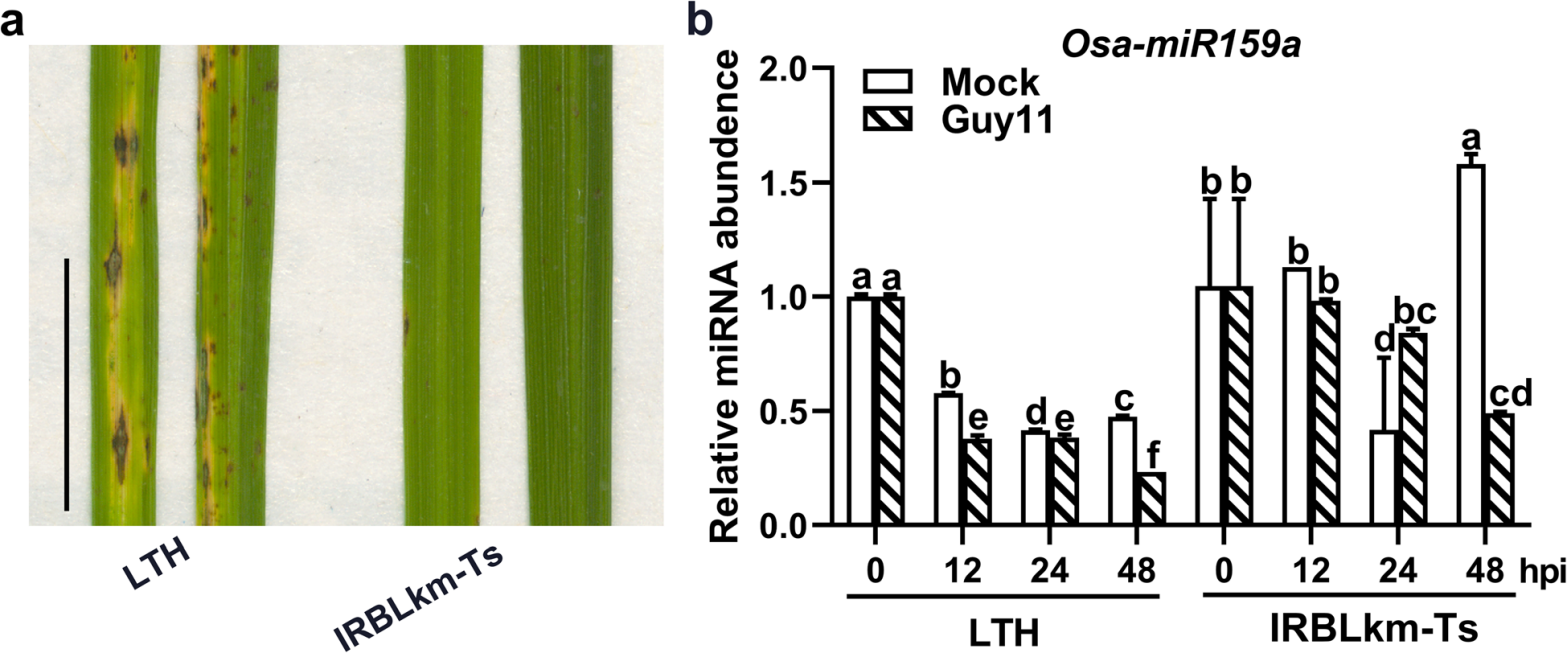
*Osa-miR159a* is differentially responsive to *Magnaporthe oryzae* in the susceptible and resistant accessions. **a** Representative leaves show the blast disease phenotype at 5 days post-inoculation (dpi) with *M. oryzae* strain Guy11 in LTH and IRBLkm-Ts. Scale bars, 10 mm. **b** Reverse transcription-quantitative PCR (RT-qPCR) shows the accumulation of *Osa-miR159a* in LTH and IRBLkm-Ts upon *M. oryzae* or mock treatment at the indicated time points. Error bars indicate SD (*n* = 3). Different letters above bars indicate significant differences (*P* < 0.05) as determined by a one-way ANOVA followed by Tukey’s HSD post hoc test. This experiment was repeated two times with similar results

Previously, the expression of *Osa-miR159a* was reported to be responsive to *M. oryzae* or its elicitors (Li et al. 2014; Li et al. 2016b; Li et al. 2019b). To confirm this conclusion, we examined its expression pattern in susceptible and resistant accessions of rice after inoculation of *M. oryzae* at the three-leaf seedling stage. The universally susceptible accession Lijiangxin Tuan Heigu (LTH) showed a severe disease phenotype, whereas the accession (IRBLkm-Ts) that contains the gene *Pikm*, which confers *M. oryzae* resistance displayed resistance (Fig. 1a). Compared with mock inoculation, *M. oryzae* infection resulted in decreased accumulation of *Osa-miR159a* at 12, 24, and 48 h post-inoculation (hpi) in LTH (Fig. 1b). In contrast, *Osa-miR159a* was significantly upregulated at 24 hpi in IRBLkm-Ts (Fig. 1b), although it was also significantly decreased at 48 hpi. These data indicated that the response of *Osa-miR159a* to *M. oryzae* infection was different in the susceptible and resistant accessions. Therefore, *Osa-miR159a* may play a role in rice immunity against *M. oryzae*.

### *Osa-miR159a* Positively Regulates Rice Resistance to *M. oryzae*

**Fig. 2:**
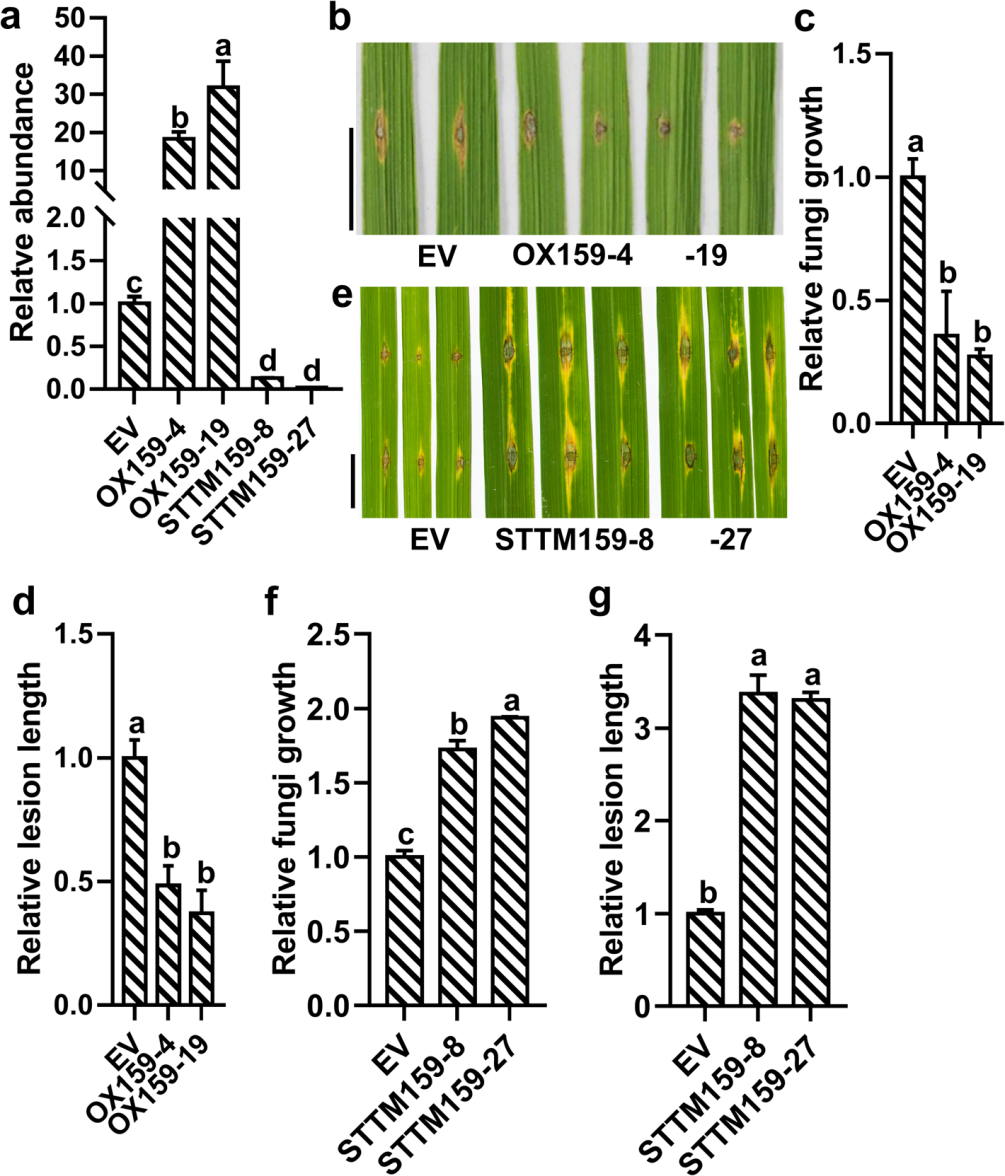
*Osa-miR159a* alleviates rice susceptibility to *Magnaporthe oryzae*. **a** Reverse transcription-quantitative PCR (RT-qPCR) data shows the relative abundance of *Osa-miR159a* in transgenic lines containing 35S: MIR159a (OX159) or transgenic lines containing STTM159 in comparison with Nipponbare (NPB) containing empty vector (EV). **b**, **e** Blast disease phenotype at 5 days post-inoculation (dpi) of *M. oryzae* strain GZ8 by punch-inoculation in the indicated lines. Scale bars, 10 mm. **c**, **f** Quantification analysis of *M. oryzae* biomass in (**b**) and (**e**), respectively. Error bars indicate SD (*n* = 3). Different letters above bars indicate significant differences (*P* < 0.05) as determined by a one-way ANOVA analysis followed by Tukey’s HSD post hoc test. **d**, **g** Relative lesion length in (**b**) and (**e**), respectively. Error bars indicate SD (*n* = 3). Different letters above bars indicate significant differences (*P* < 0.05) as determined by a one-way ANOVA analysis and Tukey’s HSD post hoc test

To explore how *Osa-miR159a* acts in the interaction between rice and *M. oryzae*, we made a construct overexpressing *Osa-miR159a* (OX159) and introduced the construct into Nipponbare (NPB), generating 24 independent transgenic lines, out of which we chose two lines that showed high *Osa-miR159a* accumulation for further investigation (Fig. 2a). We made a construct expressing STTM of *Osa-miR159a* (STTM159) and introduced it into NPB, which may prevent *Osa-miR159a* from binding to its target sites (Franco-Zorrilla et al. 2007; Todesco et al. 2010). We also selected two independent transgenic lines that showed a significant reduction of *Osa-miR159a* accumulation for further investigation (Fig. 2a). Then we conducted blast disease assays by punch- or spray- inoculation of the *M. oryzae* strain GZ8. We found that OX159 lines generated significantly smaller disease lesions than NPB harboring an empty vector (EV) (Fig. 2b and Fig. S1a). Consistently, the lesions from OX159 lines contained significantly less fungal mass and shorter lesion length than the control at 5 days post-inoculation (dpi) (Fig. 2c, d and Fig. S1b). In contrast, STTM159 lines generated significantly larger disease lesions than that of the control (Fig. 2e and Fig. S2a), and the lesions from STTM159 lines contained significantly more fungal mass and longer lesions than the control at 5 dpi (Fig. 2f, g and Fig. S2b, c). These data indicated that *Osa-miR159a* positively regulated the resistance of rice to *M. oryzae*.

Next, we exploited the GFP-tagged strain GZ8 to observe the infection process in sheath cells using laser scanning confocal microscopy. Compared with the control, our observation found that the infection progress was delayed in OX159 (Fig. S1c, d), but accelerated in STTM159 (Fig. S2d, e). At 24 hpi and 36 hpi, the percentages of invasive hyphae were much lower in OX159 (Fig. S1c, d) compared with the control; however, the percentages of invasive hyphae were greater in STTM159 (Fig. S2d, e). These results indicated that overexpressing *Osa-miR159a* delayed infection, whereas blocking *Osa-miR159a* facilitated *M. oryzae* infection.

To explain why *Osa-miR159a* positively regulated resistance to *M. oryzae*, we used RT-qPCR to examine the expressions of some marker genes, including *OsNAC4*, *OsPR10b* (*Pathogenesis-Related 1b*) and *OsJAMYB*, acting in immune responses after infection with *M. oryzae* (Park et al. 2012; Pan et al. 2014). The expression of *OsNAC4* was higher in OX159 than in the control at 6 and 12 hpi (Fig. S1e), whereas it was lower in STTM159 than in the control at 6 and 24 hpi (Fig. S2f). The expression of *OsPR10b* was higher in OX159 than in the control at 6 hpi (Fig. S1f); it was lower in STTM159 than in the control at 0, 12, and 24 hpi (Fig. S2g). The expression of *OsJAMYB* was higher in OX159 than in the control at 6 and 12 hpi (Fig. S1g), while it was lower in STTM159 than in the control at 6 and 12 hpi (Fig. S2h). These data indicated that *Osa-miR159a* activated defense-related genes, positively regulating rice resistance to *M. oryzae*.

### Alteration of *Osa-miR159a* Accumulation Leads to Developmental Defects

**Fig. 3:**
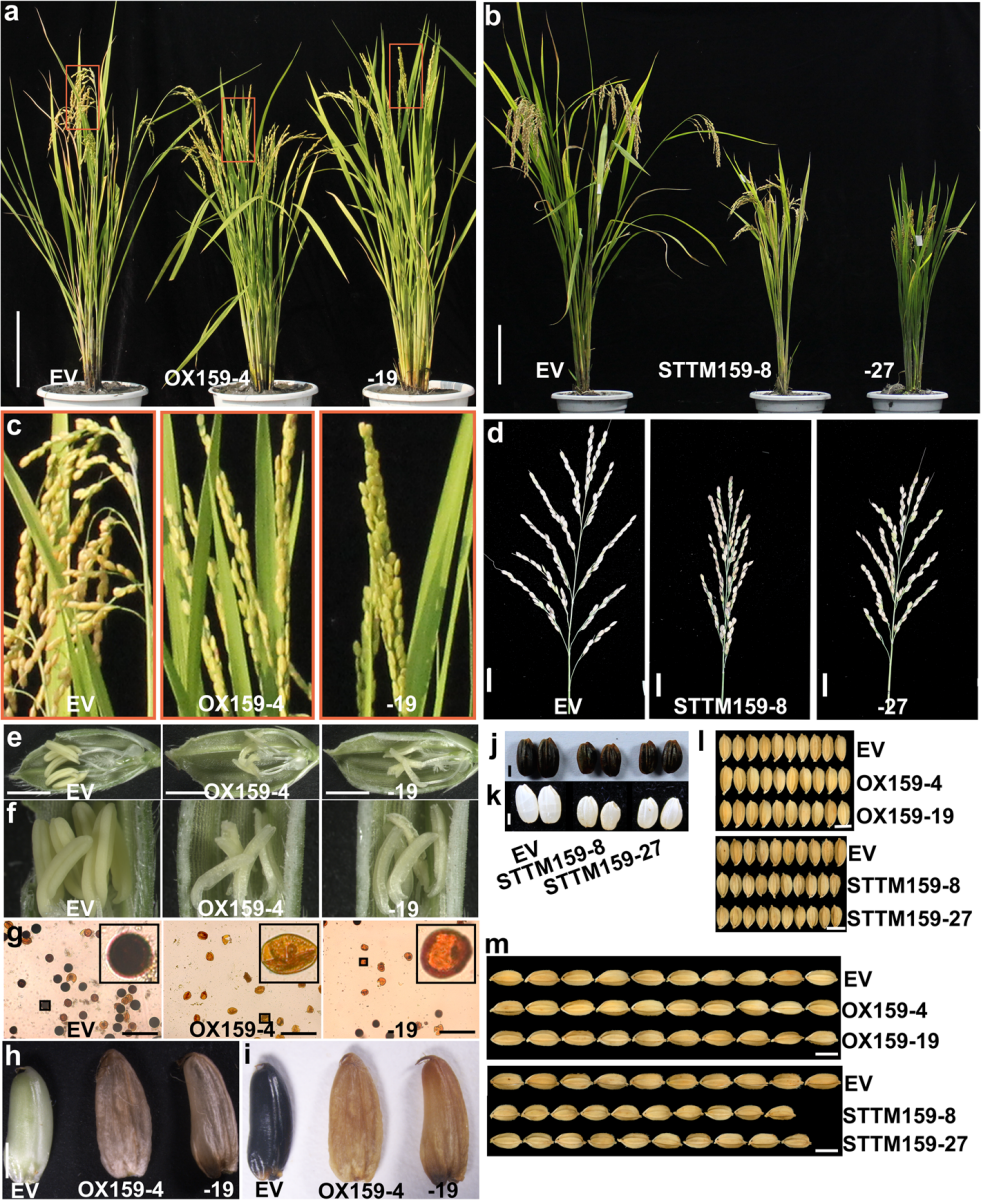
*Osa-miR159a* influences rice traits and yield. **a**, **b** Gross morphology of the indicated lines. Scale bars, 20 cm. **c**, **d** Panicles morphology of the indicated lines. Scale bars, 2 cm in (**d**). **e**, **f** Shucked grains of the indicated lines. Scale bars, 2 mm. **g** Potassium iodide dye shows the starch in the pollen grain of the indicated lines. Scale bars, 100 μm. **h**-**k** Potassium iodide dye shows the starch in seeds of the indicated lines. The pictures in (**h**, **k**) were captured before potassium iodide dye. The pictures in (**i**, **j**) were captured after potassium iodide dye. **l**, **m** Comparison of grain width (**l**) and grain length (**m**) in the indicated lines. Scale bars, 5 mm

In addition to the resistance conferred by *Osa-miR159a* in rice against *M. oryzae*, we found that both OX159 and STTM159 showed some altered development and yield traits. All the OX159 and STTM159 transgenic lines were shorter than the control (Fig. 3a, b and Table 1), with STTM159 lines significantly shorter than OX159 lines and the control (Fig. 3b and Table 1). Both OX159 and STTM159 had a lower yield (Table 1). The OX159 lines were sterile and had only a few filled grains on the panicle, leading to straight panicles at the mature stage (Fig. 3a, c and Table 1). The stamen development was deficient in OX159 lines (Fig. 3e, f). In comparison with the control, which had yellowish anthers containing fertile pollen indicated by starch-staining, anthers from OX159 were pale with sterile pollen lacking starch (Fig. 3g). In addition, grains from OX159 lines lacked starch accumulation, although the ovary grew to a size comparable to that of the control (Fig. 3h, i, l, m and Table 1). However, STTM159 showed smaller panicles than that of the control, but the starch accumulation in the grain was normal (Fig. 3d, j, k). STTM159 was also observed to be less productive than the control (Table 1). The grain width of STTM159 was the same as the control, whereas the grain length was shorter than the control (Fig. 3l, m and Table 1). These results indicated that the alteration of *Osa-miR159a* expression led to defects in growth and development, particularly in pollen and grain development.

**Table 1:** Yield traits of the wild type, OX159, and STTM159 lines grown in rice paddies^*^

Materials	Plant Height/cm	No. of Tillers	Panicle Length/cm	No. of Filled Gains Per Plant	Yield Per Plant/g	1000-grain weight/g	Grain Length/mm	Grain Width/mm
EV	95.83 ± 0.58^a^	12.80 ± 1.73^a^	19.40 ± 0.25^a^	1096.33 ± 23.50^a^	28.26 ± 0.94^a^	25.76 ± 0.49^a^	7.19 ± 0.013^b^	3.32 ± 0.02^a^
OX159-4	85.33 ± 2.08^b^	12.67 ± 1.53^a^	17.65 ± 0.35^b^	146.67 ± 106.00^d^	6.79 ± 3.53^d^	24.30 ± 0.53^b^	7.42 ± 0.082^a^	3.32 ± 0.02^a^
OX159-19	87.00 ± 1.00^b^	12.00 ± 2.65^a^	17.46 ± 0.30^b^	34.00 ± 28.48^e^	0.82 ± 0.68^e^	24.07 ± 0.13^b^	7.46 ± 0.050^a^	3.32 ± 0.012^a^
STTM159-8	58.96 ± 5.63^c^	8.20 ± 1.30^b^	15.74 ± 0.38^c^	670.00 ± 22.30^c^	12.70 ± 0.60^c^	18.97 ± 0.15^d^	6.38 ± 0.15^c^	3.34 ± 0.0074^a^
STTM159-27	66.82 ± 1.71^c^	11.00 ± 1.87^a^	15.49 ± 0.58^c^	875.80 ± 68.56^b^	19.15 ± 0.75^b^	21.90 ± 0.23^c^	6.60 ± 0.08^c^	3.34 ± 0.0017^a^

* Different letters indicate significant differences at *P* < 0.05 determined by One-way ANOVA

### Alteration of *Osa-miR159a* Expression Impacts the Expression of its Target Genes

Six *Osa-miR159* loci in rice generate five mature isoforms that share 18 central nucleotides (Fig. S3a). Among them, *Osa-miR159a/b* targeted two confirmed genes, namely, *OsGAMYB* (*LOC_Os01g59660*) and *OsGAMYBL* (*LOC_Os06g40330*) (Li et al. 2016a), and one predicated gene, *LOC_Os10g05230* (encoding a C3HC4-type domain-containing zinc finger protein, herein designated *OsZF*) (Khan et al. 2017). The target sites in *OsGAMYB* and *OsGAMYBL* were in the codon region, whereas the target site was in a 5′ untranslated region (UTR) of *OsZF* (Fig. S3b). To examine how the expression of these genes was impacted by the alteration of *Osa-miR159a* expression in OX159 and STTM159, we performed a RT-qPCR analysis. As expected, the expression of all three genes was significantly less in OX159 than in the control (Fig. S3c). In contrast, the expression of all these genes was more in STTM159 than in the control (Fig. S3c). These data indicated that the overexpression of *Osa-miR159a* significantly suppressed the expression of its target genes, and the STTM of *Osa-miR159a* prevented the suppression of *Osa-miR159a* on the expression of its target genes.

### Knockout of *OsGAMYB, OsGAMYBL*, and *OsZF* Leads to Enhanced Resistance to *M. oryzae*

**Fig. 4:**
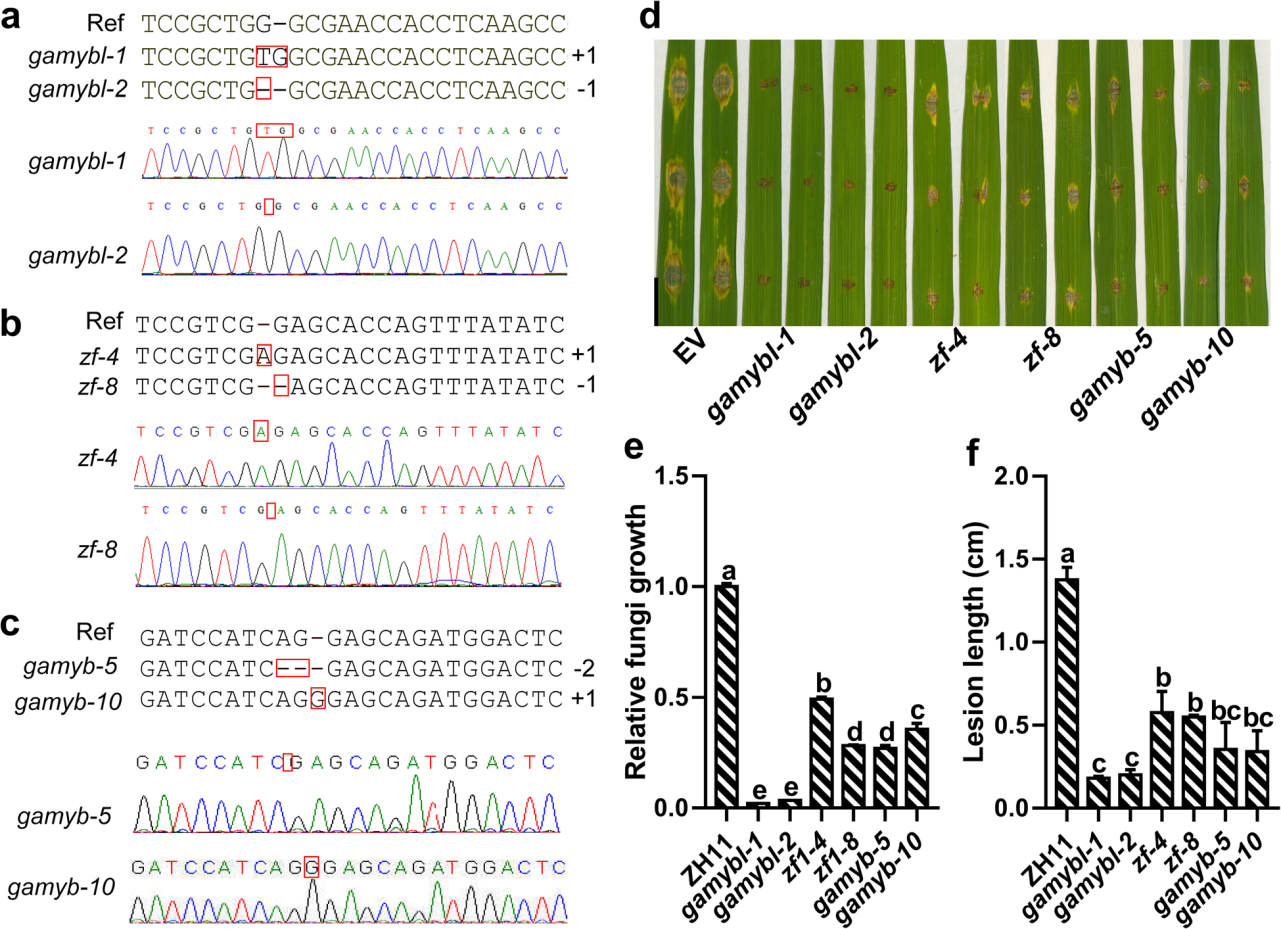
The mutations of *Osa-miR159a* target genes result in enhanced resistance against *Magnaporthe oryzae*. **a**-**c** The genotype of *Osa-miR159a* target gene knockout lines were confirmed by PCR based sequencing. Ref means reference sequences. **d** Blast disease phenotype at 5 days post-inoculation (dpi) with *M. oryzae* strain GZ8 in the indicated lines. Scale bars, 10 mm. **e**, **f** Relative fungal biomass (**e**) and lesion length (**f**) on the inoculated leaves from (**d**). Data are shown as mean ± SD (*n* = 3). Different letters above bars indicate significant differences (*P* < 0.05) as determined by a one-way ANOVA and Tukey’s HSD post hoc test. The experiments were repeated two times with similar results

To investigate the function of *OsGAMYB, OsGAMYBL*, and *OsZF*, we obtained mutants using CRISPR/Cas9 DNA editing. We identified two independent mutants for *OsGAMYBL*, *OsZF*, and *OsGAMYB*. Among them, *gamybl-1* carried a 1-bp insertion resulting in an early stop codon after aa 325 (Fig. 4a). *gamybl-2* carried a 1-bp deletion resulting in an early stop codon after aa 311 (Fig. 4a); *zf-4* carried a 1-bp insertion resulting in an early stop codon after aa 42 (Fig. 4b). The *zf-8* had a 1-bp deletion resulting in an early stop code after aa 32 (Fig. 4b); *gamyb-5* carried a 2-bp deletion resulting in an early stop codon after aa 127 (Fig. 4c). The *gamyb-10* had a 1-bp insertion resulting in an early stop codon after aa 128 (Fig. 4c). We conducted a *M. oryzae* assay via punch-inoculation. All the knockout lines significantly decreased the size of *M. oryzae* lesions that contained significantly lower fungal mass and shorter lesions than that of the control (Fig. 4d-f). Specifically, *gamybl* mutants were especially resistant than *gamyb* and *zf* mutants, indicating that *OsGAMYBL* has the most important role in disease resistance. Together, these results indicated that *OsGAMYB, OsGAMYBL*, and *OsZF* contributed to *Osa-miR159a*-mediated regulation of rice resistance to *M. oryzae*. However, based on previous studies, loss of function mutations of *OsGAMYB* resulted in shortened internodes and defects in floral organ development (Kaneko et al. 2004); loss function of *OsGAMYBL* also contribute to these phenotypes (Tsuji et al. 2006). Similarly, we found that loss of function mutations of *OsGAMYB* or *OsGAMYBL* cause defective rice development (data not shown), especially in relation to pollen development. However, the phenotype of *zf* mutants was very similar to that of the wild type, except for the slightly reduced height of the *zf* mutants (Fig. S4a), but not in 1000-grain weight (Fig. S4b). Together with the resistance phenotype, the editing of *OsZF* may have a potential application in rice breeding.

## Discussion

miRNAs act as important regulators in plant growth, development, and host-pathogen interactions (Jones-Rhoades et al. 2006; Baldrich and San Segundo 2016). Some miRNAs have been identified to be involved in fine-tuning rice resistance to *M. oryzae* and yield traits. For example, high accumulation of *Osa-miR1873* results in defects in growth and yield-related traits, and also increases susceptibility to *M. oryzae* (Zhou et al. 2020). Here, we described *Osa-miR159a*, which regulates multiple growth and yield traits, as a new positive regulator in rice resistance to *M. oryzae*. First, high accumulation of *Osa-miR159a* resulted in enhanced resistance to *M. oryzae*, which was associated with an increase in the defense response, i.e., high expression of defense-related genes (Fig. 2b-d and Fig. S1). In addition, the transgenic lines overexpressing *Osa-miR159a* showed developmental defects such as pollen sterility and grain-filling (Fig. 3a, c). However, blocking *Osa-miR159a* by STTM resulted in increased susceptibility to *M. oryzae* (Fig. 2e-g and Fig. S2). Developmental defects were also observed in STTM159 transgenic lines including shorter plants and reduced grain length (Fig. 3b, d, m). Consistent with the *M. oryzae* disease phenotypes in OX159 and STTM159, the *OsGAMYB*, *OsGAMYBL*, and *OsZF* knockout lines exhibited enhanced resistance to *M. oryzae* (Fig. 4). Therefore, *Osa-miR159a* has multiple functions in rice resistance to *M. oryzae* and rice development.

*miR159* is a conserved miRNA that represses the expression of *GAMYB-like* genes, which encode MYB domain transcription factors (Alonso-Peral et al. 2010). Proper expression of *GAMYB* and *GAMYBL* are important for rice development. GAMYB acts as a positive transcriptional regulator of GA-dependent α-amylase expression and also has important roles in floral organ development and pollen development (Kaneko et al. 2004). Here, we demonstrated that suppressing *GAMYB* by overexpressing *Osa-miR159a* resulted in sterile pollen lacking starch and failure of the grain to accumulate starch (Fig. 3e-i); however, the grain length was slightly larger than that of the wild type (Table 1). In addition, the uninhibited expression of *GAMYB* by overexpressing STTM159 also resulted in slightly shorter grains (Fig. 3m and Table 1). These results indicated that *GAMYB* was crucial for grain development. We observed that enhanced or decreased expression of *Osa-miR159a* also impacted plant height (Fig. 3a, b and Table 1). Previous studies reported that loss of function mutations of *OsGAMYB* results in shortened internodes; *OsGAMYBL* contributes to this phenotype (Kaneko et al. 2004; Tsuji et al. 2006), indicating that *OsGAMYB* and *OsGAMYBL* played a role in the height of rice plants. However, *zf* mutants showed slightly reduced height without other defects in rice development. Therefore, future studies should consider the potential application of *OsZF* in rice breeding using the CRISPR/Cas9 system.

The circadian clock and abiotic stress conditions impact gene expressions in plants (Sugiyama et al. 2001; Matsuzaki et al. 2015). Thus, mock treatments were necessary in examining the expression of *Osa-miR159a* during *M. oryzae* infection. The results showed that *Osa-miR159a* was responsive to *M. oryzae* infection compared with the mock treatment at each time point (Fig. 1). However, the mock treatment strongly influences the expression of *Osa-miR159a*, indicating that the expression of *Osa-miR159a* may be strongly impacted by the circadian clock or/and abiotic stress conditions (i.e., mock treatment), but further studies are required. In addition, our results showed that knockout lines of *OsGAMYB* and *OsGAMYBL* exhibited increased resistance to *M. oryzae* (Fig. 4). It is important to determine how *Osa-miR159a* functions in rice immunity against *M. oryzae*. On one hand, this is consistent with previous results that have shown that MYB transcription factors are involved in rice immunity. For example, the repressive MYB transcription factor, *MYBS1*, results in reduced expression of *broad-spectrum resistance-Digu 1* (*bsr-d1*) allele from the rice cultivar Digu, thus, inhibiting H_2_O_2_ degradation and enhanced disease resistance (Li et al. 2017b). *MYB30* bonds to and activates the promoter of the *4-coumarate:coenzyme A ligase* genes *Os4CL3* and *Os4CL5*, resulting in accumulation of lignin subunits G and S, further leading to obvious thickening of sclerenchyma cells and inhibiting *M. oryzae* penetration (Li et al. 2020b). *BGIOSGA004670*, the homolog of *GAMYB* in *Arabidopsis*, showed increased expression upon fungal infection, suggesting that *GAMYB-like* genes might be involved in resistance to fungal infection (Li et al. 2016b). The novel MYB transcription factor *CaPHL8* acts as a positive regulator in the resistance of pepper to *Ralstonia solanacerum* (Noman et al. 2019). On the other hand, *GAMYB* is involved in GA-signaling; thus, *GAMYB* may regulate rice immunity by manipulating plant hormones. Moreover, loss-of-function of *ZF* also results in enhanced resistance to *M. oryzae*. Zinc finger proteins are involved in plant growth and development. Overexpression of *zinc finger protein 1* (*GhZFP1*) enhances resistance to *Rhizoctonia solani* (Guo et al. 2009). In the future, we will focus on the function of *ZF* in the resistance of rice immunity to *M. oryzae*.

An increasing number of studies have shown that the production of RNAi-inducing dsRNA in the host can result in specific fungal gene silencing, further conferring resistance to fungal pathogens (Zhang et al. 2016b). In response to *Verticillium dahlia* infection, cotton plants increase *miR166* and *miR159* expression, and export them to fungal hyphae for silencing key genes that are essential for fungal virulence (Zhang et al. 2016b). Hence, we cannot exclude the possibility that *Osa-miR159a* may also be exported to the fungal hyphae of *M. oryzae* to silence genes that are essential for fungal virulence. Future studies are required to confirm this hypothesis.

## Conclusions

We functionally characterized *Osa-miR159a* and its target genes in rice resistance to *M. oryzae*. Our data indicated that *Osa-miR159a* positively regulated resistance to *M. oryzae* and impacted yield traits by regulating its target genes *OsGAMYB*, *OsGAMYBL*, and *OsZF*. Suppressed expression of *OsGAMYB*, *OsGAMYBL*, and *OsZF* by overexpressing *Osa-miR159a* or knockout of *OsGAMYB*, *OsGAMYBL*, and *OsZF* resulted in enhanced resistance to *M. oryzae*, but led to developmental defects. In contrast, blocking *Osa-miR159a* via STTM led to significantly increased susceptibility and defects in yield traits. Therefore, proper spatiotemporal expression of *Osa-miR159a* was critical for rice immunity and development. *Osa-miR159a* and the regulatory module of its target genes could be used to breed rice with resistance to *M. oryzae*.

## Materials and Methods

### Plant Materials and Growth Conditions

*Oryza sativa Japonica* accessions Nipponbare (NPB) and Zhong Hua 11 (ZH11) were used for transgenic analysis. The susceptible accession Lijiangxin Tuan Heigu (LTH) and the resistant accession International Rice Blast Line Pyricularia-Kanto51-m-Tsuyuake (IRBLkm-Ts) was used in this study. The rice plants were grown in a growth chamber maintained at 26 °C and 70% relative humidity under 12 h of light and 12 h of darkness. To assay yield traits, rice plants were grown in a paddy field in the Wenjiang district of Chengdu, China, from April to September.

### Analysis of Yield Traits

Rice agronomic traits were measured from five plants in the paddy field at maturity. The 1000-grain weight, grain length, and grain width were measured using an SC-A grain analysis system (Wanshen Ltd., Hangzhou, China) using the filled grains that were dried in an oven at 42 °C for 1 week. These data were analyzed by a one-way ANOVA followed by post hoc Tukey’s HSD analysis. Differences were considered significant at *P* < 0.01.

### Plasmid Construction and Genetic Transformation

To construct the transgenic line overexpressing *Osa-miR159a*, we amplified the DNA sequence containing 321 bp upstream and 306 bp downstream of *Osa-miR159a* from NPB genomic DNA with the specific primers miR159a-*Kpn*I-F and miR159a-*Sal*I-R (Table S1), then the amplified DNA fragments were digested and cloned into the binary vector 35S-pCAMBIA1300 at *Kpn*I and *Sal*I sites, resulting in the overexpressing construct. To construct the transgenic lines overexpressing the short tandem target mimic (STTM) of *Osa-miR159a* (STTM159), we inserted the amplified DNA fragments of STTM159 into the *Kpn*I and *Sal*I sites of the binary vector 35S-pCAMBIA1300, resulting in a target mimic of the miR159 construct. The entire sequences of STTM159 were 5′-ggtaccTGCAGCTCCTGATCGGGCATGCAAGTTGTTGTTGTTATGGTCTAGTTGTTGTTGTTATGGTCTAATTTAAATATGGTCTAAAGAAGAAGAATATGGTCTAAAGAAGAAGAATCAGAGCTCCCTCAGTCAATCCAAAgtcgac-3′. Both of the constructs were transformed into the NPB background via *Agrobacterium*-mediated transformation. To generate *Osa-miR159a* target gene knockout lines, we constructed the CRISPR/Cas9 plasmids as described previously (Zhao et al. 2019). The constructs were transformed into the ZH11 background via *Agrobacterium* strain GV3101. All the positive transgenic lines were confirmed using hygromycin. To confirm the genotype of the knockout lines, we performed PCR-based gene sequencing as described previously (Zhao et al. 2019). All primers are listed in Table S1.

### Pathogen Infection and Microscopy Analysis

*Magnaporthe oryzae* strains Guy11 and eGFP-tagged Zhong10-8-14 (GZ8) was used in this study. The strain was incubated on oatmeal and tomato agar media (OTA) at 28 °C under a 12-h light and 12-h dark cycle. After 10 days, the hyphae were scratched, and the fungus on the plates was further incubated with 24-h light treatment to promote sporulation. Three to 5 days later, the spores were collected for spray- or punch-inoculation. For spray-inoculation, seedlings at the three-leaf-stage were inoculated with a spore suspension (3 × 10^5^ conidia/mL). Disease lesions were recorded at 5 days post-inoculation (dpi). For punch-inoculation, 5 μL of the spore suspension (5 × 10^5^ spore/mL) was drop-inoculated at wound sites on the leaves of seedlings at the three-leaf-stage following previously described methods (Li et al. 2014). Briefly, the dilution-drop conidia suspension was placed against wounded sites on the leaves. Lesion formation was examined at 4–6 dpi. The relative fungal mass was calculated using the DNA concentration of *M. oryzae Pot2* against the rice genomic *Ubiquitin* DNA level by quantitative PCR.

*M. oryzae* strain GZ8 was used to observe the fungal infection process. Leaf sheaths (5-cm-long) were inoculated with a spore suspension (1 × 10^4^ conidia/mL) as described previously (Li et al. 2014). The inoculated epidermal layer was excised for observation. We observed the invasion process including appressorium development and invasive hyphal growth with a Laser Scanning Confocal Microscope (Nikon A1) at 24-, 36-, and 48- hpi. The quantitative analysis of the infestation stage was conducted as described previously (Li et al. 2017a).

### Reverse Transcription Quantitative Polymerase Chain Reaction (RT-qPCR)

The leaves of OX159 and STTM159 were collected to detect the amounts of miRNA and target genes. To examine the expressions of defense-related genes, we inoculated seedlings in the three-leaf-stage with *M. oryzae* using the spray inoculation method. The inoculated leaf samples were collected at 0, 6, 12, and 24 hpi. Total RNA was extracted, then reverse transcription was performed following a previous report (Zhao et al. 2021). To analyze the expression of miRNA, we performed a stem-loop pulse RT-qPCR following a previous report (Turner et al. 2013). U6 snRNA was used as an internal reference to normalize data.

## Supplementary Information

**Additional file 1 **: **Figure S1.** Overexpression of *Osa-miR159a* results in enhanced resistance to *Magnaporthe oryzae*. **a** Blast disease phenotype at 5 days post-inoculation (dpi) with *M. oryzae* strain GZ8. Scale bars, 10 mm. **b** Relative lesion area on the inoculated leaves from (**a**). Error bars indicate standard deviation (SD) (*n* = 3). Asterisks (^**^) above the bar indicates significant differences (*P* < 0.01) determined by the Student’s *t*-test. **c** Confocal images showing the infection status of eGFP-tagged *M. oryzae* strain GZ8 in the indicated lines at 24, 36, and 48 h post-inoculation (hpi). Scale bars, 25 μm. **d** Quantification analysis on the process of GZ8 infection in the indicated lines at the indicated time points. Over 200 conidia in each line were analyzed. The experiments were repeated two times with similar results. **e**-**g** The expression of defense-related genes in wild type and OX159 lines following the inoculation of *M. oryzae* strain GZ8. RNA was extracted at the indicated time points for the reverse transcription-quantitative PCR (RT-qPCR) assay. The amounts of collected mRNA were normalized to that in the wild type at 0 hpi. Error bars indicate SD (*n* = 3). Different letters above the bars indicate significant differences (*P* < 0.01) as determined by a one-way ANOVA followed by post hoc Tukey’s HSD analysis.

**Additional file 2 **: **Figure S2.** Overexpression of STTM159 results in enhanced susceptibility to *Magnaporthe oryzae*. **a** Blast disease phenotype at 5 days post-inoculation (dpi) with *M. oryzae* strain GZ8. Scale bars, 10 mm. **b**, **c** Relative fungal growth and lesion area on the inoculated leaves from (**a**). Error bars indicate SD (*n* = 3). Asterisks (^**^) above the bar indicates significant differences (*P* < 0.01) determined by the Student’s *t*-test. **d** Confocal images showing the infection status of a GFP-tagged *M. oryzae* strain GZ8 in the indicated lines at 24, 36, and 48 h post-inoculation (hpi). Scale bars, 25 μm. **e** Quantification analysis of the process of GZ8 infection in the indicated lines at the indicated time points. Over 200 conidia in each line were analyzed. The experiments were repeated two times with similar results. **f**-**h** The expression of defense-related genes in wild type (WT) and STTM159 lines following the inoculation of *M. oryzae* strain GZ8. RNA was extracted at the indicated time points for the reverse transcription-quantitative PCR (RT-qPCR) assay. The amounts of collected mRNA were normalized to that in the WT at 0 hpi. Error bars indicate SD (*n* = 3). Different letters above the bars indicate significant differences (*P* < 0.01) as determined by a one-way ANOVA followed by post hoc Tukey’s HSD analysis.

**Additional file 3 **: **Figure S3.** *Osa-miR159* mature isoforms and the accumulation of *Osa*-*miR159a* target genes in the indicated lines. **a** The sequence alignments of *Osa*-*miR159* mature isoforms and their positions on the chromosome in rice. **b** The structure of target genes and the sequence alignment of the target sites in the target genes. White boxes indicate the 5′-UTRs and 3′-UTRs. Black boxes indicate exons. Black lines indicate introns. Red lines indicate the target sites of *Osa-miR159a*. **c** Reverse transcription-quantitative PCR (RT-qPCR) data show the relative mRNA amount of target genes in OX159 and STTM159 in comparison with NPB containing the empty vector (EV). Data are shown as mean ± SD (*n* = 3). Different letters above bars indicate significant differences (*P* < 0.05) as determined by a one-way ANOVA followed by post hoc Tukey’s HSD analysis.

**Additional file 4 **: **Figure S4.** The phenotype and 1000-grain weight (g) of the *zf* mutants. **a** The phenotype of *zf* mutants and EV control at the reproductive stage. The height of *zf* mutants slightly less than that of the wild type plants. **b** The 1000-grain weight (g) of the indicated plants showed no difference.

**Additional file 5 **: **Table S1.** The primers used in this research.

## Data Availability

All of the datasets are included within the article and its additional files.

## Abbreviations

STTM: Short tandem target mimic
ARF: Auxin response factor
LTH: Lijiangxin Tuan Heigu
IRBLkm-Ts: International Rice Blast Line Pyricularia-Kanto51-m-Tsuyuake
NPB: Nipponbare
ZH11: Zhong Hua 11
CRISPR: Clustered regularly interspaced short palindromic repeats
OTA: Tomato agar media
RT-qPCR: Reverse transcription quantitative polymerase chain reaction
LSCM: Laser scanning confocal microscopy
TCP: PROLIFERATING CELL NUCLEAR ANTIGEN BINDING FACTOR
