# Supplementary figures and images for: Fine-Tuning Roles of Osa-miR159a in Rice Immunity Against Magnaporthe oryzae and Development
Source: Rice (N Y). 2021 Mar 6;14:26. doi: 10.1186/s12284-021-00469-w (PMC7937009; doi:10.1186/s12284-021-00469-w)

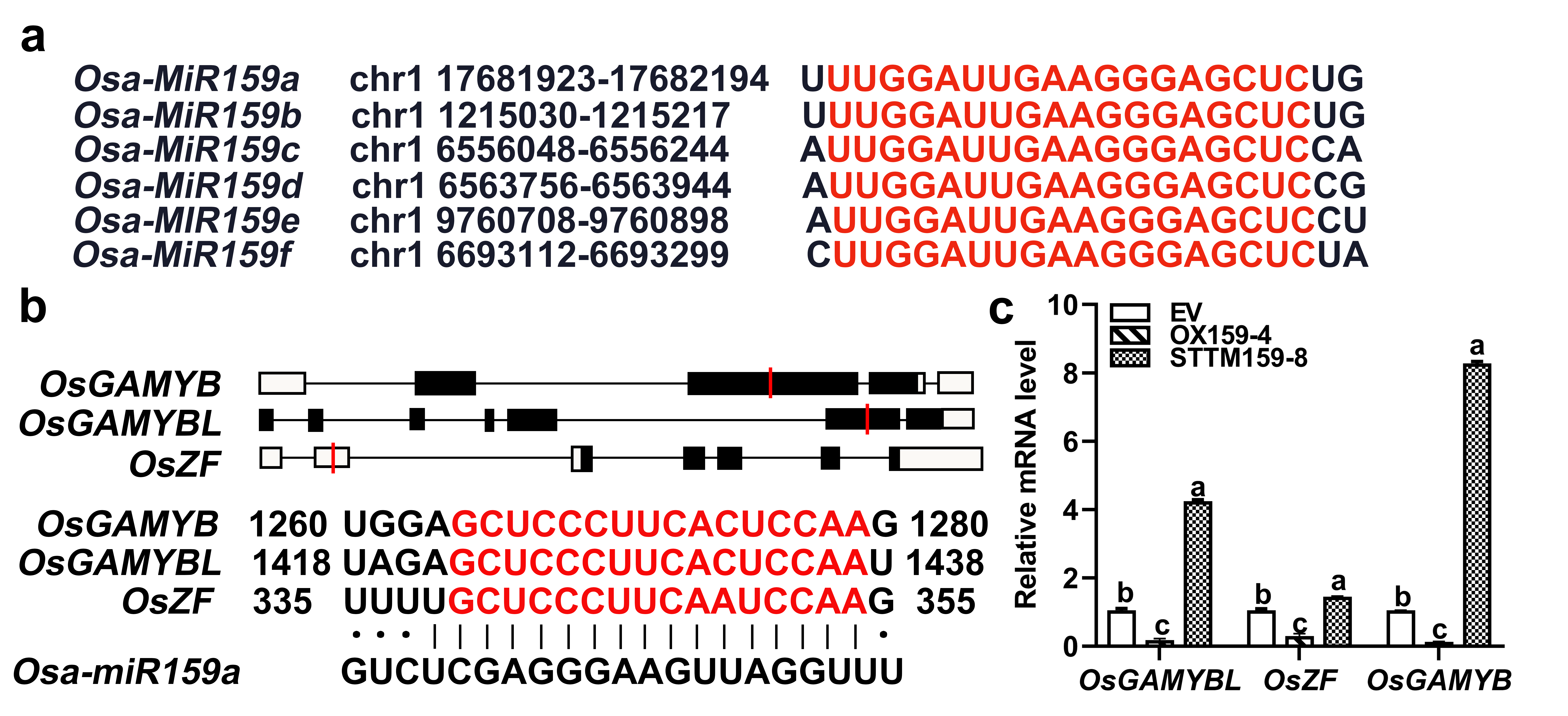

Supplement: Supplementary file 3 — Additional file 3 : Figure S3. Osa-miR159 mature isoforms and the accumulation of Osa-miR159a target genes in the indicated lines. a The sequence alignments of Osa-miR159 mature isoforms and their positions on the chromosome in rice. b The structure of target genes and the sequence alignment of the target sites in the target genes. White boxes indicate the 5′-UTRs and 3′-UTRs. Black boxes indicate exons. Black lines indicate introns. Red lines indicate the target sites of Osa-miR159a. c Reverse transcription-quantitative PCR (RT-qPCR) data show the relative mRNA amount of target genes in OX159 and STTM159 in comparison with NPB containing the empty vector (EV). Data are shown as mean ± SD (n = 3). Different letters above bars indicate significant differences (P < 0.05) as determined by a one-way ANOVA followed by post hoc Tukey’s HSD analysis. [file 12284_2021_469_MOESM3_ESM.tif]

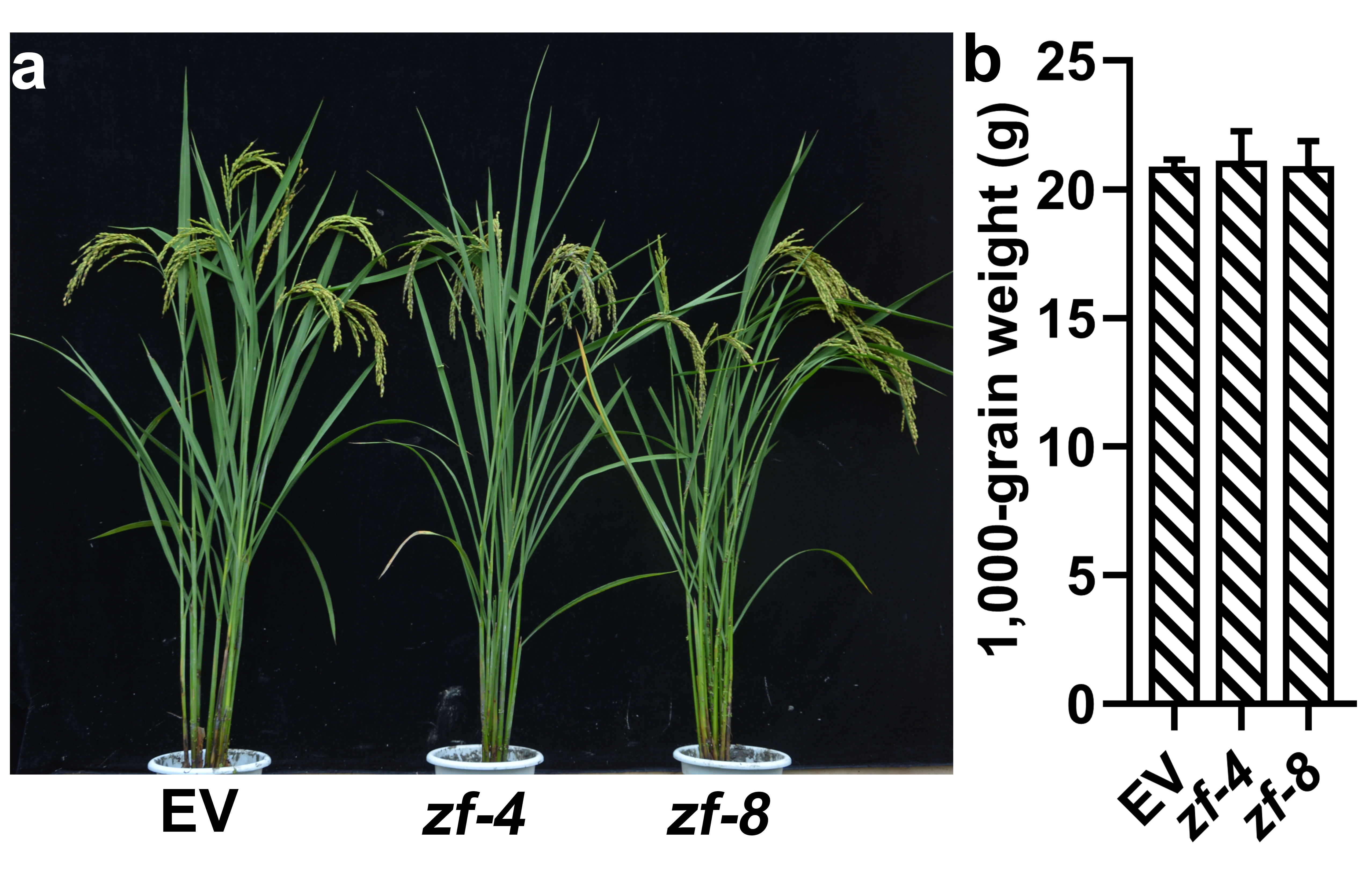

Supplement: Supplementary file 4 — Additional file 4 : Figure S4. The phenotype and 1000-grain weight (g) of the zf mutants. a The phenotype of zf mutants and EV control at the reproductive stage. The height of zf mutants slightly less than that of the wild type plants. b The 1000-grain weight (g) of the indicated plants showed no difference. [file 12284_2021_469_MOESM4_ESM.tif]
