# Supplementary material for: Fine-Tuning Roles of Osa-miR159a in Rice Immunity Against Magnaporthe oryzae and Development
Source: Rice (N Y). 2021 Mar 6;14:26. doi: 10.1186/s12284-021-00469-w (PMC7937009; doi:10.1186/s12284-021-00469-w)
Supplement: Supplementary file 5 — Additional file 5 : Table S1. The primers used in this research. [file 12284_2021_469_MOESM5_ESM.docx]

**Table S1. The primers used in this research**

| Primer name | Sequence (5’-3’) | Annotation |
| --- | --- | --- |
| Osa-miR159a stem-loop | GTCGTATCCAGTGCAGGGTCCGAGGTATTCGCACTGGATACGACCAGAGC | For miR159a accumulation |
| Osa-miR159a-F | CGGGCTTTGGATTGAAGGG | RT-qPCR |
| Universal primer-R | CAGTGCAGGGTCCGAGGTAT | RT-qPCR |
| U6-F | CGATAAAATTGGAACGATACAGA | RT-qPCR |
| U6-R | ATTTGGACCATTTCTCGATTTGT | RT-qPCR |
| OsGAMYBL-F | TTCCTTGACAGCCAACCTCC | RT-qPCR |
| OsGAMYBL-R | GCGTCATCTGCATCTTTGGC | RT-qPCR |
| OsZF-F | GGATGTTTGCCCAATCTGTCT | RT-qPCR |
| OsZF-R | GGGCAAGTTTCACTCCGCT | RT-qPCR |
| OsGAMYB-F | TCACTGAATCCACCCCTCCT | RT-qPCR |
| OsGAMYB-R | GGTGAAGTGTCCCCAGGTTC | RT-qPCR |
| UBQ-F | GCCCAAGAAGAAGATCAAGAAC | RT-qPCR |
| UBQ-R | AGATAACAACGGAAGCATAAAAGTC | RT-qPCR |
| MoPot2_F | ACGACCCGTCTTTACTTATTTGG | RT-qPCR |
| MoPot2_R | AAGTAGCGTTGGTTTTGTTGGAT | RT-qPCR |
| OsNAC4-F | TCCTGCCACCATTCTGAGATG | RT-qPCR |
| OsNAC4-R | TTGCAGAATCATGCTTGCCAG | RT-qPCR |
| OsJAMYB-F | GACCTCACCCTCATCAATTAC | RT-qPCR |
| OsJAMYB-R | AGCTCTTCCCAGTCCTCTTC | RT-qPCR |
| OsPR10b-F | TGAGGTGTTGGAGGTTAAGAG | RT-qPCR |
| OsPR10b-R | AGGGTGAGCGACGAGGTA | RT-qPCR |
| miR159a-*Kpn*1-F | ACAGGTACCTTGTGAAGATTCGAGCCAACC | For cloning |
| miR159a-*Sal*1-R | ACAGTCGACAAGAGGAAGGATAAGCATGG | For cloning |
| STTM159a-*Kpn*1-F | GGTACCTGCAGCTCCTGATCGGGCATGCAA | For cloning |
| STTM159a-*Sal*1-R | GTCGACTTTGGATTGACTGAGGGAGCTCTG | For cloning |
| *gamybl*-F | GACGCCCGACGAGGACAA | For sequencing |
| *gamybl*-R | GCAAAACCAAGGAACCAT | For sequencing |
| *gamyb*-F | ACTCGGCTTCTCGTCATTGC | For sequencing |
| *gamyb*-R | GCCGTGCTTCTTCACGTAGT | For sequencing |
| *zf*-F | ACTGTTTTTGTGTAGGTCTTTGAGA | For sequencing |
| *zf*-R | TGGCTTTCATCTAACTTGCAGC | For sequencing |
